# Molecular sensitization profiles and β-parvalbumin sensitization burden associated with clinical fish allergy in children

**DOI:** 10.3389/fimmu.2026.1918831

**Published:** 2026-08-03

**Authors:** Sevda Tuten Dal, Lala Heydarova, Bulent Enis Sekerel

**Affiliations:** 1 Department of Pediatric Allergy, Hacettepe University Faculty of Medicine, Ankara, Türkiye; 2 Department of Pediatrics, Azerbaijan State Advanced Training Institute for Doctors named after Aziz Aliyev, Baku, Azerbaijan

**Keywords:** fish allergy, molecular allergology, pediatric allergy, sensitization burden, β-parvalbumin

## Abstract

**Background:**

Fish allergy is a potentially severe condition that is frequently managed with broad elimination diets. Molecular allergology may improve risk stratification by distinguishing clinically relevant fish allergy from asymptomatic sensitization. We aimed to characterize molecular sensitization patterns in fish-sensitized children and identify molecular profiles associated with clinical fish allergy.

**Methods:**

Sixty-one fish-sensitized children (0–18 years) were evaluated using a multiplex molecular allergy assay (ALEX2). Twenty-eight children were classified as fish-allergic based on a positive oral food challenge (OFC) or consistent clinical reactions, whereas 33 sensitized children were clinically tolerant. Molecular sensitization patterns were analyzed using Bayesian logistic model averaging (BMA), Firth penalized logistic regression, receiver operating characteristic (ROC) analysis, and hierarchical clustering.

**Results:**

Sensitization to β-parvalbumins was significantly more frequent in allergic than tolerant children. Allergic children also exhibited a substantially higher cumulative β-parvalbumin sensitization burden (median 7 [IQR 5–7] vs 3 [IQR 1–5]; p<0.001). BMA prioritized Clu h 1, Gad m 2_3, and Cyp c 1 as the molecular allergens most strongly associated with clinical fish allergy. Sensitization to Gad m 2_3 was observed exclusively among allergic children. An exploratory parsimonious model incorporating cumulative β-parvalbumin sensitization burden together with Gad m 2_3 achieved an optimism-corrected AUC of 0.867.

**Conclusions:**

Molecular sensitization profiles, particularly the cumulative burden of β-parvalbumin sensitization, were associated with clinical fish allergy in children. Clu h 1 and Cyp c 1 emerged as the molecular markers most consistently associated with clinical reactivity, whereas Gad m 2_3 was observed only among allergic children in this cohort and may represent a low-prevalence exploratory finding. These findings suggest a potential role for molecular allergen profiling in risk stratification among fish-sensitized children but require validation in larger independent cohorts before clinical application.

## Introduction

Fish allergy is relatively uncommon in childhood, with an estimated prevalence of 0.4% in the United States and a challenge-confirmed prevalence of approximately 0.1% in Europe; in both settings, it is observed more often in older children than in early childhood (1, 2). In Türkiye, population-based prevalence data are lacking. However, clinic-based cohorts indicate that fish allergy is rare in infancy (0.4% in children aged 0–2 years) and becomes more frequent in older children and adolescents (2.4% in those aged 3–18 years) (3, 4).

Fish constitutes an important component of childhood nutrition, providing high-quality protein and long-chain omega-3 fatty acids that are essential for growth and neurodevelopment. Despite these benefits, fish allergy remains one of the more challenging immunoglobulin E (IgE)-mediated food allergies in childhood, owing to its frequent persistence into later life and its association with potentially severe reactions, including anaphylaxis (5, 6). In routine clinical practice, children diagnosed with fish allergy are commonly advised to avoid all fish species. This conservative strategy may result in extensive dietary limitations and negatively affect nutritional adequacy and quality of life, particularly during periods of growth and development. However, accumulating evidence suggests that this generalized avoidance strategy may be unnecessarily restrictive for a substantial proportion of fish-sensitized children (7, 8).

From a molecular standpoint, fish allergy is predominantly associated with sensitization to β-parvalbumin, a low-molecular-weight, heat-stable calcium-binding muscle protein that is widely distributed among bony fish species (9). Although β-parvalbumins share considerable structural homology, substantial interspecies variability exists with respect to parvalbumin content and isoform composition (10, 11). These quantitative differences have been proposed as a key factor underlying the observed discrepancy between extensive IgE cross-reactivity at the serological level and the more limited degree of clinically relevant cross-reactivity seen *in vivo* (10, 12). Consequently, a subset of fish-sensitized individuals may tolerate specific fish species despite detectable parvalbumin-specific IgE (10, 12, 13).

Advances in molecular allergology, including component-resolved diagnostics, have enabled a more refined assessment of sensitization to fish molecular allergens, revealing heterogeneous sensitization patterns among fish-sensitized patients (9, 14). Recent studies have demonstrated that sensitization to individual fish molecular allergens does not uniformly translate into clinical allergy and that the qualitative pattern of sensitization may be more informative than sensitization to a single allergen alone (8, 12, 13). Nevertheless, distinguishing clinically relevant sensitization from asymptomatic sensitization remains challenging, particularly in pediatric populations, due to the diversity of fish species, limitations of skin prick testing, and the practical constraints of oral food challenges (OFCs).

In this context, a more detailed characterization of sensitization profiles to fish molecular allergens and their relationship with clinical reactivity is needed.

The present study aimed to evaluate molecular sensitization patterns in fish-sensitized children using a multiplex molecular allergy approach and to identify sensitization profiles associated with clinical fish allergy. We further sought to explore whether individual molecular sensitizations and the cumulative burden of β-parvalbumin sensitization could help distinguish clinically allergic children from sensitized but tolerant individuals and thereby support more individualized risk assessment and dietary management strategies.

## Materials and methods

### Study population

This study was performed at the Pediatric Allergy Division of Hacettepe University Faculty of Medicine. Between 1 February 2020 and 3 August 2024, molecular allergen sensitization patterns were assessed in 740 pediatric patients (aged 0–18 years) using a commercial multiplex array (ALEX2, Macro Array Diagnostics, Vienna, Austria) in two independent laboratories blinded to the clinical characteristics of the patients. The study population comprised patients with a reported history of allergic reactions to any fish species and/or documented sensitization to at least one fish allergen molecule detected by the ALEX2 test.

Sensitization to the following fish molecular allergens was recorded: Sco s 1, Thu a 1, Cyp c 1, Clu h 1, Sal s 1, Xip g 1, Gad m 1, Gad m 2_3, and Raj c parvalbumin. The nomenclature and biochemical classification of these fish molecular allergens are provided in **Table 1** and were verified according to the World Health Organization and International Union of Immunological Societies (WHO/IUIS) Allergen Nomenclature Sub-Committee (15), with ALEX2 platform nomenclature retained where appropriate.

**Table 1:** Fish allergen molecules examined in the study.

Species (english name)	Allergen	Biochemical name
Scomber scombrus (Atlantic mackerel)	Sco s 1	Beta-parvalbumin
Thunnus albacares (Tuna)	Thu a 1	Beta-parvalbumin
Cyprinus carpio (Carp)	Cyp c 1	Beta-parvalbumin
Clupea harengus (Herring)	Clu h 1	Beta-parvalbumin
Salmo salar (Salmon)	Sal s 1	Beta-parvalbumin
Xiphias gladius (Swordfish)	Xip g 1	Beta-parvalbumin
Gadus morhua (Atlantic cod)	Gad m 1	Beta-parvalbumin
Gadus morhua (Atlantic cod)	Gad m 2	Beta-enolase
Gadus morhua (Atlantic cod)	Gad m 3	Aldolase A
Raja clavata (Thornback ray)	Raj c parvalbumin	Alpha-parvalbumin

Gad m 2 and Gad m 3 are listed separately in the WHO/IUIS database but are reported as the combined component Gad m 2_3 in the ALEX2 platform. Raj c parvalbumin represents the α-parvalbumin of Raja clavata and follows ALEX2 nomenclature, consistent with the fish allergy literature.

Patients and parents were interviewed face-to-face or by telephone regarding their age at first fish consumption, history of clinical reactivity to any bony fish species, and current fish consumption patterns. Given that cartilaginous fish such as ray are not commercially produced, marketed, or consumed in Türkiye, the assessment of clinical reactivity was restricted to bony fish species. The clinical information provided by the patients was cross-checked with the records of emergency and allergy units. The diagnoses of allergic rhinitis, asthma, and atopic dermatitis were made according to international guidelines (16–18). Patients were classified as fish-allergic if they had a history of at least two consistent clinical reactions—with at least one confirmed by physician assessment or visual evidence—or a positive OFC. Individuals who were sensitized but lacked both a history of consumption and a desire to introduce fish into their diet were excluded from the study.

### Oral food challenge protocol

Open oral food challenges (OFCs) were performed according to the standardized protocol described in the Food Challenge Tests: Turkish National Guideline 2020, which is based on the PRACTALL consensus recommendations (19, 20). Challenges were performed with an individual fish species and were therefore species-specific. The most frequently used challenge species were sea bass, sea bream, salmon, and tuna. Incremental doses were administered at approximately 20-minute intervals, beginning with 5 mg of fish and increasing logarithmically. If no reaction occurred during the incremental phase, an age-appropriate meal-sized portion of the same fish species was subsequently administered over 30–60 minutes, resulting in a cumulative intake of approximately 97 g of fish (corresponding to approximately 20 g of fish protein). The challenge was discontinued and considered positive when objective immediate allergic manifestations consistent with IgE-mediated hypersensitivity occurred. A negative OFC was interpreted as evidence of tolerance only to the fish species used in that challenge and not to all fish species.

### In vitro assays

The ALEX2 multiplex microarray test (21) (marketed as a macroarray) encompasses 178 molecular allergens and 117 extracts (22). Within this array, allergens are immobilized on a nitrocellulose membrane within a cartridge chip, and the employed diluent contains a cross-reactive carbohydrate determinant (CCD) inhibitor with an inhibition efficiency of at least 85%. The results of molecular allergen sensitizations are presented as kilo–allergen units per liter (kUA/L), ranging from 0.3 to 50 kUA/L.

For the purposes of this study, sensitization was defined as an allergen-specific IgE concentration ≥0.30 kUA/L. Values below this threshold were considered negative for binary sensitization analyses and were not treated as missing values. Quantitative allergen-specific IgE values were used only for descriptive comparisons among sensitized participants and were not analyzed across the entire study cohort.

### Statistical analysis

Continuous variables were summarized as median and interquartile range (IQR) because their distributions were non-normal. Between-group comparisons for continuous variables were performed using the Mann–Whitney U test. Categorical variables were compared using Fisher’s exact test, while the Fisher–Freeman–Halton exact test was used for multi-category age-group comparisons. To account for multiple allergen-level comparisons, p values were adjusted using the Benjamini–Hochberg false-discovery-rate (FDR) procedure. These allergen-level analyses were considered exploratory.

The cumulative β-parvalbumin sensitization burden was calculated as the number of positive β-parvalbumin components among Gad m 1, Cyp c 1, Clu h 1, Sal s 1, Sco s 1, Thu a 1, and Xip g 1. Gad m 2_3 and Raj c were not included in this burden score because Gad m 2_3 corresponds to the combined ALEX2 component representing Gad m 2 (β-enolase) and Gad m 3 (aldolase A), whereas Raj c represents α-parvalbumin.

To evaluate the relative contribution of molecular allergens while accounting for model uncertainty, Bayesian logistic model averaging (BMA) was performed using all nine binary molecular sensitization indicators. The complete model space consisted of 512 candidate models (2^9^). Posterior inclusion probabilities (PIPs) were calculated for each allergen and used for exploratory variable prioritization. BMA was not used to estimate effect sizes; odds ratios were derived from regression models.

Because Gad m 2_3 sensitization occurred exclusively among allergic children, standard maximum-likelihood logistic regression was avoided owing to complete separation. Firth penalized logistic regression was used to estimate odds ratios (ORs), 95% confidence intervals (CIs), and profile-likelihood p values. The primary parsimonious model included two predictors (approximately 14 events per parameter), whereas the BMA-prioritized molecular model included three predictors (approximately 9.3 events per parameter). Model discrimination was assessed using receiver operating characteristic (ROC) analysis and area under the curve (AUC) values with bootstrap 95% confidence intervals. Internal validation was performed using 1,000 bootstrap resamples to estimate optimism-corrected AUC values. Classification performance was summarized using sensitivity, specificity, positive and negative predictive values, positive and negative likelihood ratios (LR+ and LR−), and Youden’s J index. The cutoff maximizing Youden’s J was considered an exploratory candidate threshold rather than a clinically validated decision threshold. An additional sensitivity analysis was performed among participants with OFC-confirmed diagnoses only. Predicted probabilities generated by the parsimonious model developed in the full cohort were applied to the OFC-confirmed subgroup without redeveloping the prediction model.

Similarity among binary molecular sensitization indicators was assessed using phi correlation coefficients. Hierarchical clustering was performed using Jaccard distance and average linkage. Bootstrap cluster support was calculated to evaluate cluster stability. Correlation and clustering analyses were interpreted as co-sensitization patterns rather than direct evidence of immunological cross-reactivity.

Statistical analyses were performed using IBM SPSS Statistics version 25.0, Python 3.13 with SciPy and scikit-learn, and custom scripts for Bayesian logistic model averaging and Firth penalized logistic regression. Statistical significance was defined as a two-sided p value <0.05. For allergen-level analyses, FDR-adjusted p values were reported.

## Results

### Patient characteristics

A total of 61 children were included, all sensitized to at least one fish allergen molecule on the ALEX2 test. Among them, 28 patients (46%) were fish-allergic based on clinical history and/or positive OFC, while 33 patients (54%) were sensitized but tolerated fish ingestion. OFC was performed in 20 of 28 (71.4%) fish-allergic children, confirming clinical allergy, and in 26 of 33 (78.8%) sensitized but tolerant children, confirming clinical tolerance. The remaining eight fish-allergic children fulfilled the predefined clinical diagnostic criteria based on a history of at least two consistent immediate reactions to fish, with at least one reaction confirmed by physician assessment or contemporaneous visual evidence. Objective immediate allergic manifestations documented in the medical records included physician-documented anaphylaxis in three children and physician-documented urticaria and/or angioedema in five children. All children classified as sensitized but tolerant reported regular fish consumption without any history of clinical reactions at the time of study inclusion. Detailed information about the fish species associated with clinical reactions, oral food challenges, and fish consumption patterns is provided in **Supplementary Table 1**.

Age and sex distributions were comparable between the allergic and sensitized but tolerant groups, with no significant differences observed across age categories (**Table 2**).

**Table 2:** Characteristics of the study population.

Characteristic	Total n:61	Sensitized but tolerant n:33	Fish-allergic n:28	*p* value
Sex (male), n (%)	48 (78.7)	23 (69.7)	25 (89.3)	0.115
Age (year), median (IQR)	4.6 (3.2-8.5)	4.6 (2.7-8.5)	4.8 (3.4-7.8)	0.908
Age(month) at first fish consumption, median (IQR)	12 (11-13)	11 (10-12)	12 (12-18)	0.009
Age subgroup, n (%)				1.000
1–5 years	39 (63.9)	21 (63.6)	18 (64.3)	
6–11 years	14 (23.0)	8 (24.2)	6 (21.4)	
12–18 years	8 (13.1)	4 (12.1)	4 (14.3)	
Number of beta-parvalbumin allergen molecule sensitization median (IQR)	5 (2-7)	3 (1-5)	7 (5-7)	<0.001
Sco s 1 n (%) median sIgE* (IQR)	44 (72.1)	18 (54.5)	26 (92.9)	0.003
	2.3 (0.9-8.9)	0.9 (0.5-1.5)	6 (2.1-20.3)	0.001
Thu a 1 n (%) median sIgE* (IQR)	43 (70.5)	19 (57.6)	24 (85.7)	0.027
	2.1 (0.7-6.3)	0.6 (0.4-1.9)	5.8 (1.5-24.1)	0.001
Cyp c 1 n (%) median sIgE* (IQR)	39 (63.9)	14 (42.4)	25 (89.3)	<0.001
	2.2(0.9-8.1)	0.9 (0.6-1.2)	3.7 (2-21.2)	0.003
Clu h 1 n (%) median sIgE* (IQR)	38 (62.3)	12 (36.4)	26 (92.9)	<0.001
	3.1(1-9.9)	1.2 (0.7-2.6)	5(1.8-19.6)	0.018
Sal s 1 n (%) median sIgE* (IQR)	38 (62.3)	14 (42.4)	24 (85.7)	0.002
	2.4 (1-12.1)	1.1(0.5-1.7)	4.5(1.7-20.6)	0.006
Xip g 1 n (%) median sIgE* (IQR)	36 (59)	14 (42.4)	22 (78.6)	0.013
	3 (0.8-16.8)	1 (0.5-2.6)	4.9 (1.8-17.4)	0.022
Gad m 1 n (%) median sIgE* (IQR)	24 (39.3)	8 (24.2)	16 (57.1)	0.022
	1 (0.7-3)	0.7 (0.4-0.9)	1.3 (0.9-8.5)	0.022
Gad m 2_3 n (%) median sIgE* (IQR)	6 (9.8)	0	6 (21.4)	—
	1.2 (0.7-4.8)		1.2 (0.7-4.8)	
Raj c parvalbumin n (%) median sIgE* (IQR)	6 (9.8)	1 (3.0)	5 (17.9)	0.085
	0.6(0.5-2.7)	0.6(0.6-0.6)	1.6 (0.4-2.9)	1.000
Current asthma, n (%)	30 (49.2)	11 ( 33.3)	19 (67.9)	0.038
Current atopic dermatitis, n (%)	19 (31.1)	13 (39.4)	6 (21.4)	0.205
Current allergic rhinitis, n (%)	38 (62.3)	16(48.5)	22(78.6)	0.038
Current food allergy, n (%)	55 (90.2)	28 (84.8)	27 (96.4)	0.205
Total IgE (kU/L), median, (IQR)	597 (236-1416)	800 (282- 1756)	370 (224-868)	0.138

*kUA/L.

P values for allergen-level positivity and quantitative sIgE comparisons were adjusted using the Benjamini–Hochberg false discovery rate (FDR) procedure. P values for clinical comorbidities were adjusted separately within the clinical-comorbidity family. All other p values are unadjusted.

IgE, immunoglobulin E; IQR, interquartile range; kU/L, kilo–units per liter; kUA/L, kilo–allergen units per liter; sIgE, allergen-specific immunoglobulin E.

The age at first fish consumption was significantly higher in the allergic group compared with sensitized but tolerant group (median 12 [IQR 12–18] vs 11 [IQR 10–12] months; p=0.009). Total serum IgE levels did not differ significantly between groups. Current asthma and current allergic rhinitis were more frequent among allergic children after FDR adjustment, whereas current atopic dermatitis and current food allergy did not differ significantly between groups (**Table 2**).

Co-sensitization to other food and aeroallergens was common in the study population. Among food allergens, sensitization most frequently involved tree nuts (87.9%) and peanut (67.2%), whereas sensitization to aeroallergens was predominantly observed for tree (60.6%) and grass (52.4%) pollens (**Table 3**).

**Table 3:** Other accompanying molecular allergen sensitizations.

Allergen	n(%)	Allergen	n(%)
Tree nuts	53 (87.9)	Fruit	14 (23.0)
Peanut	41 (67.2)	Tree pollen	37 (60.6)
Legumes	40 (65.5)	Grass pollen	32 (52.4)
Seed	33 (54.1)	Weed pollen	31 (50.8)
Cereals	13 (21.3)	House Dust Mite	13(21.3)
Egg	26 (42.6)	Storage Mite	7 (11.5)
Milk	18 (29.5)	Mould	17 (27.9)
Shellfish	3(4.9)	Malassezia	5 (8.2)
Meat	3 (4.9)	Cockroach	8 (13.1)

### Sensitization profile

Sensitization to β-parvalbumin components, including Gad m 1, Cyp c 1, Clu h 1, Sal s 1, Sco s 1, Thu a 1, and Xip g 1, was generally more frequent and associated with higher allergen-specific IgE levels in children with clinical fish allergy than in sensitized but tolerant children (**Table 2**). After FDR adjustment, positivity to all β-parvalbumin components remained significantly associated with fish allergy, whereas Raj c showed no significant between-group difference (**Table 2**). Sensitization to Gad m 2_3 was detected in six children, all of whom belonged to the allergic group (FDR-adjusted p=0.012).

When the cumulative number of β-parvalbumin sensitizations was evaluated using the corrected seven-component burden score, allergic children exhibited a substantially higher sensitization burden than tolerant children (median 7 [IQR 5–7] vs 3 [IQR 1–5]; total cohort median 5 [IQR 2–7]; p<0.001) (**Table 2**). In this cohort, a broader β-parvalbumin sensitization profile was associated with clinical fish allergy in addition to sensitization to individual molecular components.

### Bayesian logistic model averaging and regression models

**Figure 1:**
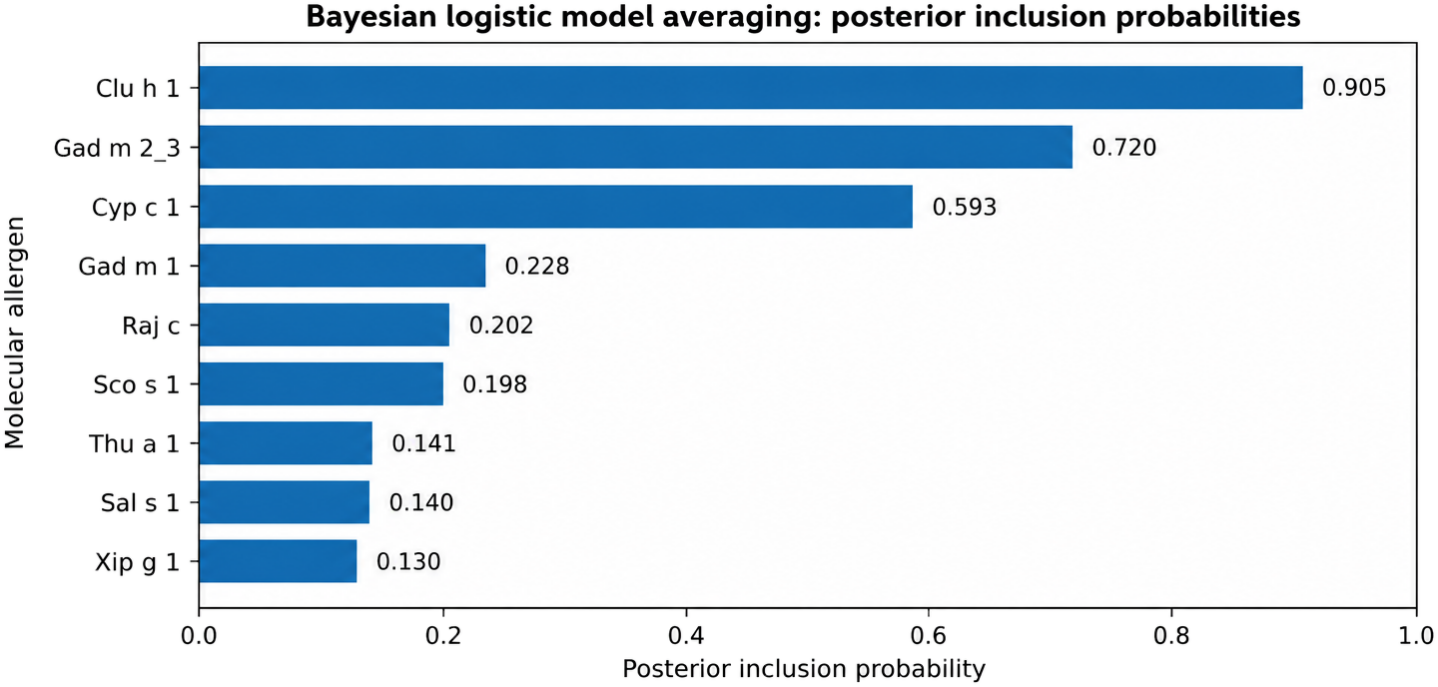
Posterior inclusion probabilities from Bayesian logistic model averaging (BMA) performed in the full study cohort (n = 61). All nine binary molecular sensitization indicators were entered into the complete model space (2^9^ = 512 candidate models). Posterior inclusion probabilities (PIPs) represent the probability that each molecular allergen is included across the posterior model distribution. Higher PIP values indicate greater relative importance for distinguishing children with clinical fish allergy from sensitized but tolerant children. The analysis was used for exploratory variable prioritization rather than confirmatory effect estimation.

To identify the molecular allergens most strongly associated with clinical fish allergy while accounting for model uncertainty, Bayesian logistic model averaging (BMA) was performed using all nine binary molecular sensitization indicators and the complete model space of 512 candidate models. Clu h 1 showed the highest posterior inclusion probability (PIP = 0.905), followed by Gad m 2_3 (PIP = 0.720) and Cyp c 1 (PIP = 0.593). The remaining allergens showed substantially lower PIPs: Gad m 1 (0.228), Raj c (0.202), Sco s 1 (0.198), Thu a 1 (0.141), Sal s 1 (0.140), and Xip g 1 (0.130). The highest posterior probability model included Clu h 1, Gad m 2_3, and Cyp c 1. These findings were interpreted as measures of variable importance and exploratory prioritization rather than confirmatory effect estimates (**Figure 1**).

To quantify the association between molecular sensitization patterns and clinical fish allergy, Firth penalized logistic regression was performed. This method was preferred over standard logistic regression because Gad m 2_3 sensitization occurred exclusively among allergic children, resulting in complete separation. Each additional β-parvalbumin sensitization was associated with increased odds of clinical fish allergy (OR 1.84, 95% CI 1.37–2.66, p<0.001). Sensitization to Gad m 2_3 was associated with a high point estimate (OR 12.11), although the confidence interval was wide (95% CI 0.94–1767.80), reflecting the small number of positive cases and complete separation. Accordingly, Gad m 2_3 should be interpreted as an exploratory candidate marker observed only among allergic children in this cohort rather than as an independently validated predictor.

### Diagnostic performance

**Figure 2:**
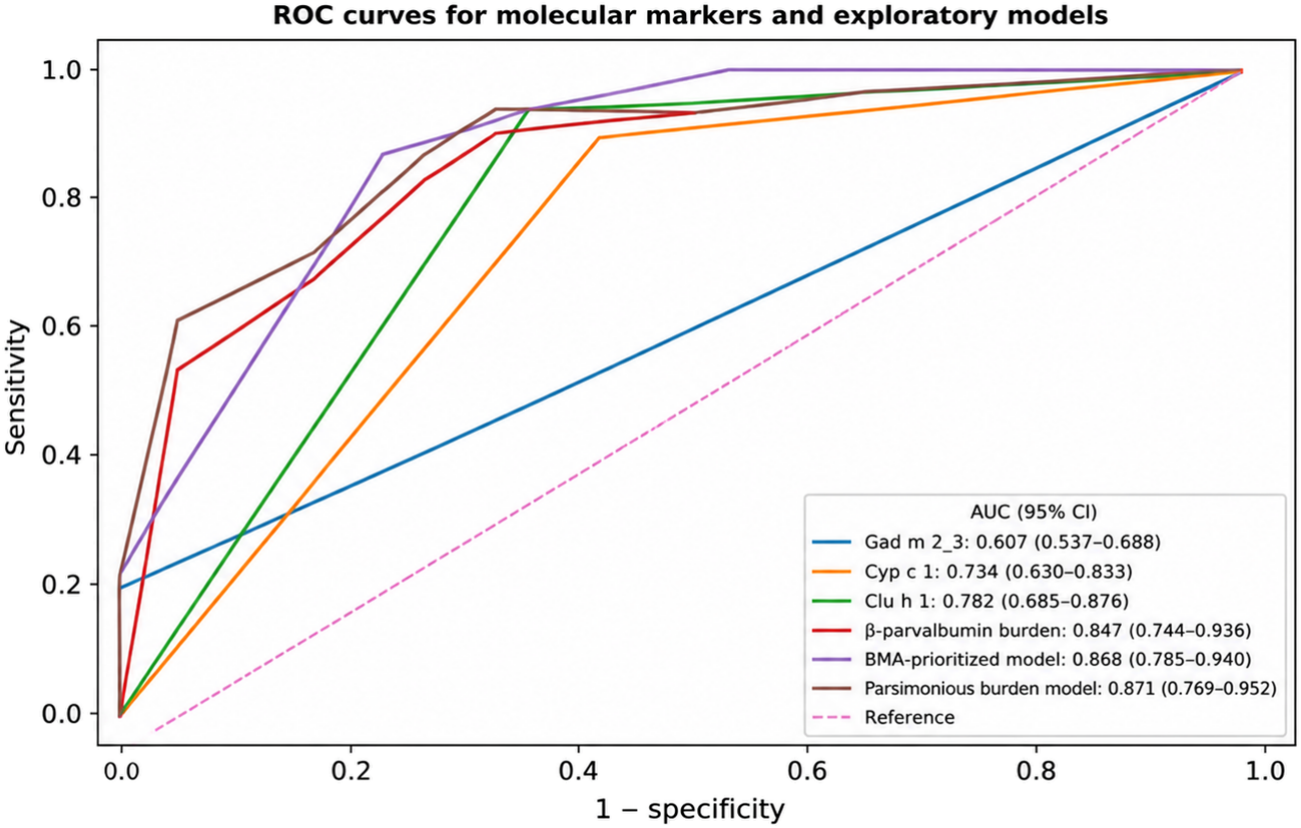
Receiver operating characteristic (ROC) curves for individual molecular markers, the cumulative B-parvalbumin sensitization burden, the Bayesian logistic model averaging (BMA)-prioritized molecular model (Clu h 1, Cyp c 1, and Gad m 2_3), and the parsimonious burden model (cumulative β-parvalbumin burden and Gad m 2_3) in the full study cohort (n = 61). Area under the curve (AUC) values with 95% bootstrap confidence intervals (CIs) are displayed in the legend. Model-based AUC values are apparent estimates obtained in the study cohort.

To evaluate the discriminatory performance of individual molecular allergens and multivariable sensitization models for identifying clinical fish allergy, ROC analyses were performed. The parsimonious model including cumulative β-parvalbumin sensitization burden and Gad m 2_3 achieved an apparent AUC of 0.871 (bootstrap 95% CI 0.769–0.952) and an optimism-corrected AUC of 0.867 after 1,000 bootstrap resamples. The molecular model based on BMA-prioritized allergens (Clu h 1, Cyp c 1, and Gad m 2_3) showed similar performance, with an apparent AUC of 0.868 (bootstrap 95% CI 0.785–0.940) and an optimism-corrected AUC of 0.860. Individual AUCs were 0.607 for Gad m 2_3, 0.734 for Cyp c 1, 0.782 for Clu h 1, and 0.847 for cumulative β-parvalbumin sensitization burden (**Figure 2**).

**Figure 3:**
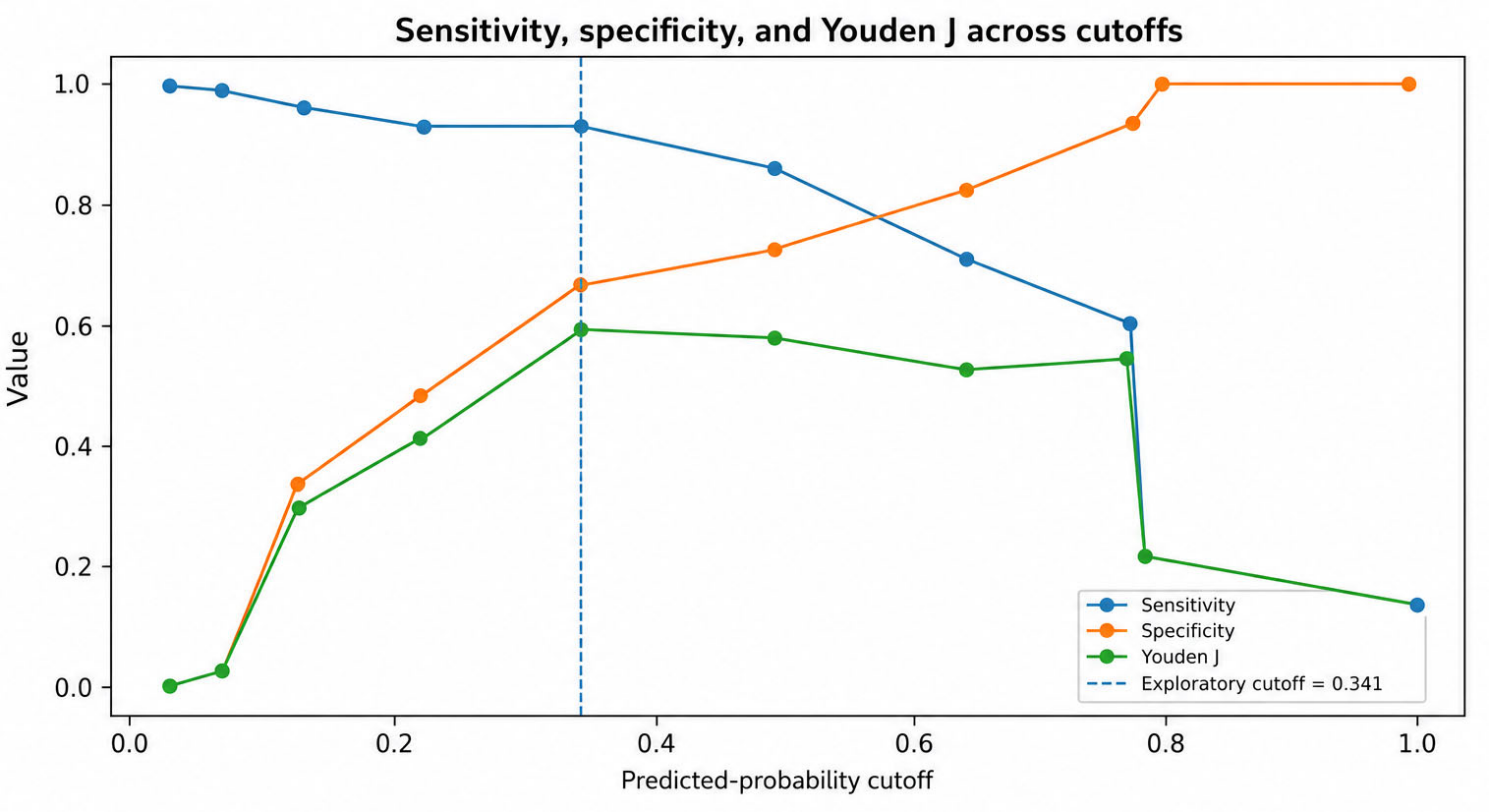
Sensitivity, specificity, and Youden's J index across predicted-probability cutoffs for the parsimonious burden model in the full study cohort (n = 61). The dashed vertical line indicates the exploratory cutoff maximizing Youden's J index (0.341). This threshold was derived and evaluated in the same cohort and should not be interpreted as a clinically validated decision threshold.

To explore potential decision thresholds for risk stratification, classification performance was evaluated across predicted-probability cutoffs using Youden’s J index. At the exploratory Youden cutoff of 0.341 for the parsimonious burden model, sensitivity was 92.9%, specificity was 66.7%, positive predictive value was 70.3%, negative predictive value was 91.7%, LR+ was 2.79, and LR− was 0.11. At the conventional 0.50 cutoff, sensitivity decreased to 71.4%, whereas specificity increased to 81.8%. Because the Youden cutoff was both derived and evaluated within the same cohort, it should be regarded as an exploratory candidate threshold requiring external validation (**Figure 3**). To further evaluate the robustness of the parsimonious model, an additional sensitivity analysis was performed in participants with OFC-confirmed diagnoses only.

### Sensitivity analysis restricted to OFC-confirmed participants

To assess the potential impact of outcome misclassification, a sensitivity analysis was performed including only participants with OFC-confirmed diagnoses (20 fish-allergic and 26 sensitized but tolerant children). Rather than redeveloping the prediction model in this substantially smaller subgroup, the predicted probabilities generated by the parsimonious model developed in the full cohort were applied to the OFC-confirmed subgroup. The model maintained comparable discriminatory performance, with an apparent AUC of 0.848 (95% CI, 0.720–0.949). Using the previously identified exploratory cutoff of 0.341, sensitivity was 90.0%, specificity 69.2%, positive predictive value 69.2%, negative predictive value 90.0%, LR^+^ 2.93, and LR^-^ 0.14 (**Supplementary Figure 1**).

### Correlation and clustering

**Figure 4:**
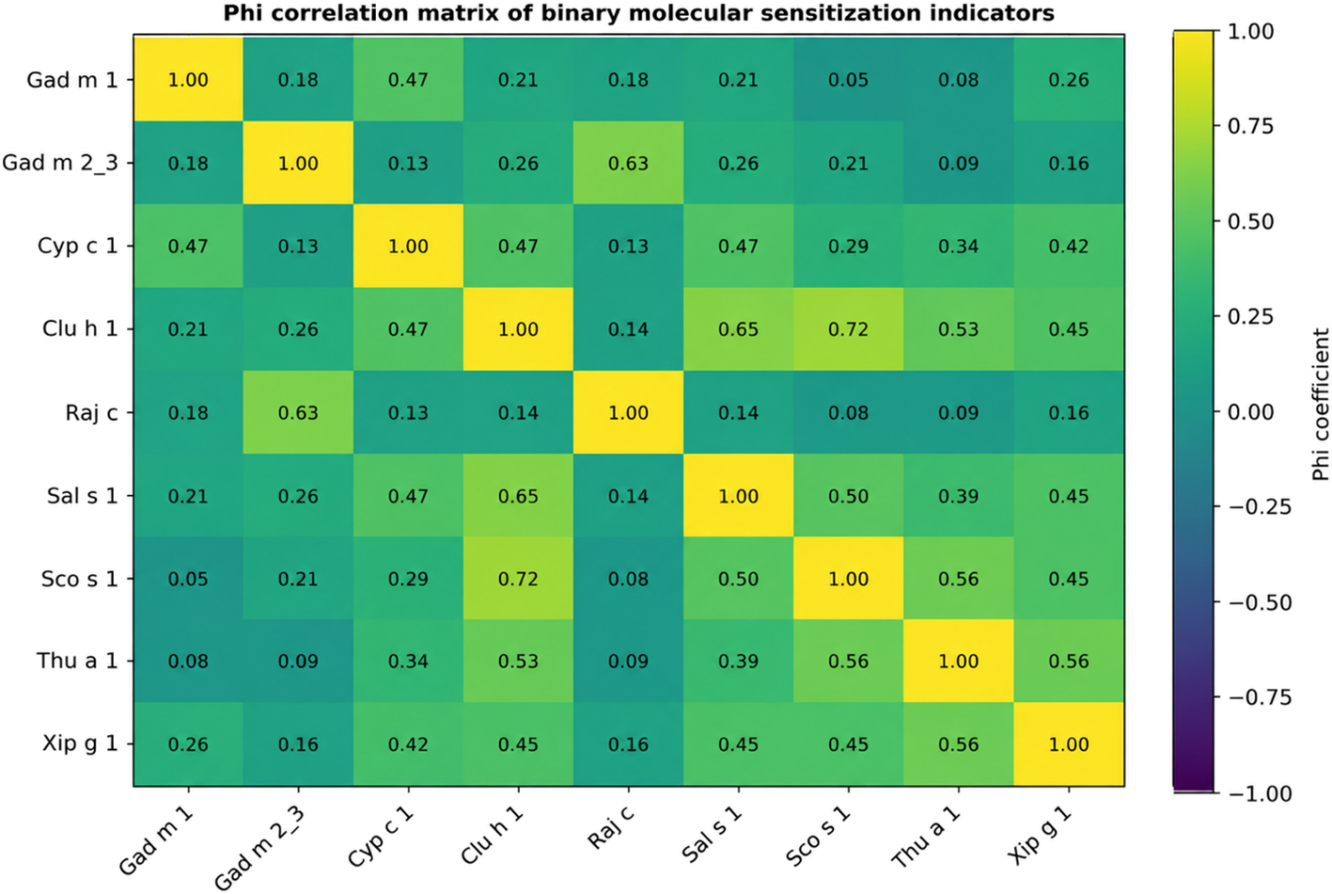
Phi correlation matrix of binary molecular sensitization indicators in the full study cohort (n = 61). Higher positive phi coefficients indicate more closely related co-sensitization patterns. These associations are compatible with, but do not establish, immunological cross-reactivity.

To explore relationships among fish molecular allergen sensitizations beyond individual prevalence analyses, pairwise associations between binary sensitization indicators were evaluated using phi correlation coefficients. This approach was used to identify patterns of co-sensitization among molecular allergens and to determine whether specific allergens tended to occur together within the same individuals. Phi correlation analysis demonstrated moderate-to-strong positive associations among several β-parvalbumin sensitizations, particularly between Clu h 1 and Sco s 1 (phi coefficient=0.72), Clu h 1 and Sal s 1 (0.65), and Sco s 1 and Thu a 1 (0.56) (**Figure 4**). These findings indicate closely related co-sensitization patterns among β-parvalbumins. However, correlation analyses alone cannot establish immunological cross-reactivity, which would require inhibition studies, structural or sequence homology analyses, or functional validation.

**Figure 5:**
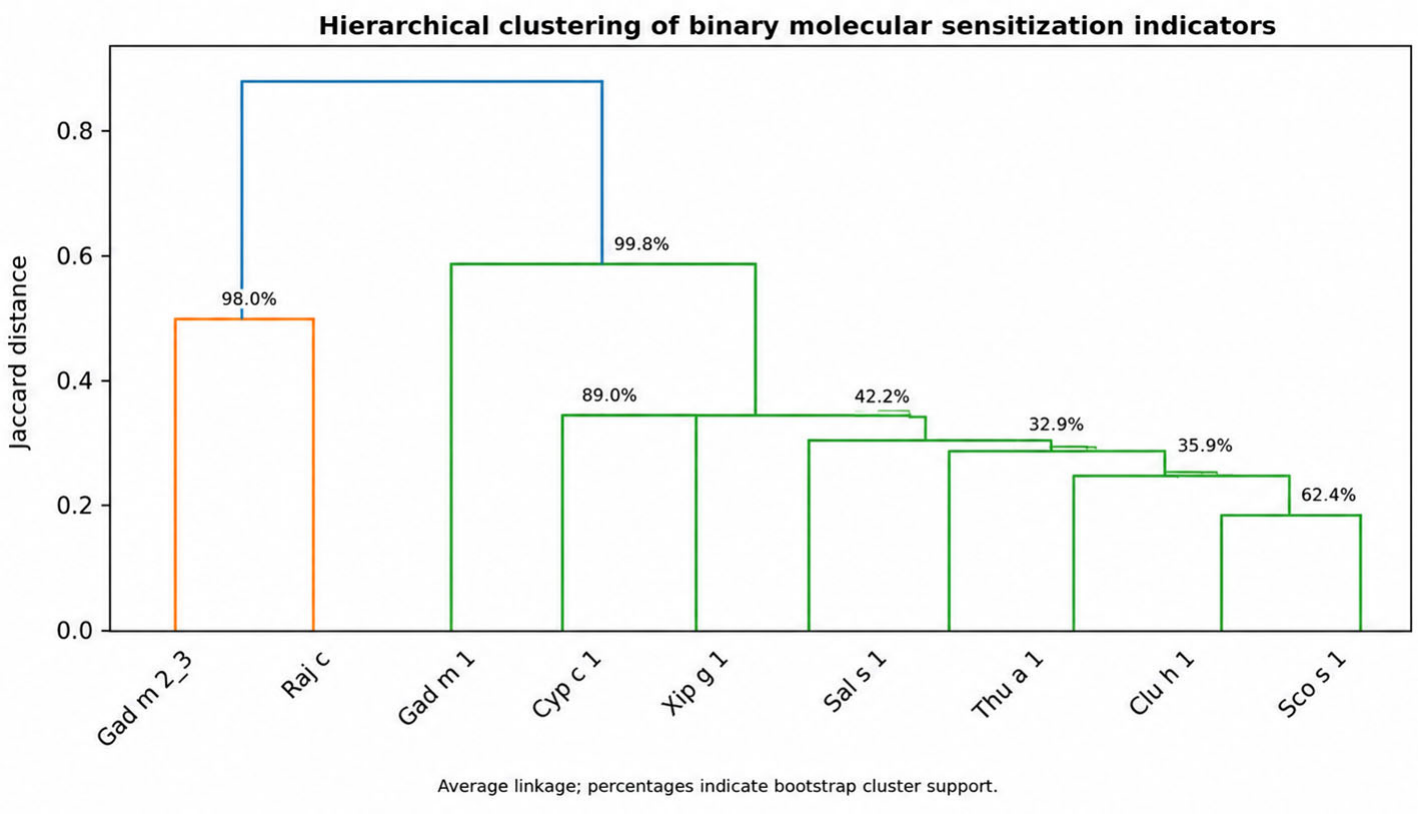
Hierarchical clustering dendrogram of binary molecular sensitization indicators in the full study cohort (n = 61) based on Jaccard distance and average linkage. Percentages shown at the internal nodes represent bootstrap cluster support values. The dendrogram summarizes similarity in co-sensitization patterns and should not be interpreted as evidence of immunological cross-reactivity.

To further investigate the overall structure of molecular sensitization patterns, hierarchical clustering was performed using Jaccard distance and average linkage. The resulting dendrogram identified a closely related cluster of β-parvalbumin sensitizations that was largely consistent with the phi correlation structure (**Figure 5**). Clu h 1, Sco s 1, Thu a 1, Sal s 1, and Xip g 1 formed the most closely related cluster, whereas Gad m 2_3 and Raj c showed distinct clustering patterns. Bootstrap support values generally indicated stable higher-level cluster structure. These findings should be interpreted as patterns of co-sensitization similarity rather than direct evidence of immunological cross-reactivity.

## Discussion

In this study, our findings suggest that molecular sensitization patterns to fish allergens provide clinically relevant information beyond the presence of fish-specific IgE alone. While sensitization to fish is frequently detected in pediatric practice, sensitization does not uniformly translate into clinical allergy, highlighting the need for more refined approaches to distinguish clinically relevant sensitization from asymptomatic sensitization (7, 13). In our cohort, both the qualitative profile of molecular sensitization and the cumulative burden of β-parvalbumin sensitization were associated with clinical fish allergy, supporting the potential value of molecular allergen profiling for risk stratification in fish-sensitized children.

Among the evaluated fish molecular allergens, sensitization to Clu h 1 and Cyp c 1 was most consistently associated with clinical fish allergy. These sensitizations showed both significantly higher prevalence and markedly higher median sIgE levels in allergic children, consistent with previous component-resolved diagnostic studies identifying β-parvalbumins as major fish allergens with high clinical relevance (10, 11, 14). In addition, sensitization to other β-parvalbumin–containing fish allergens, including Sco s 1, Thu a 1, Sal s 1, Xip g 1, and Gad m 1, was also significantly more prevalent and quantitatively stronger in allergic children. Taken together, these findings suggest that clinical fish allergy is associated with a broader pattern of β-parvalbumin sensitization rather than sensitization to a single dominant molecular allergen.

Importantly, when the cumulative number of sensitized β-parvalbumin molecules was evaluated, children with clinical fish allergy exhibited a markedly higher sensitization burden than sensitized but tolerant children (p<0.001). This finding suggests that the breadth of β-parvalbumin sensitization, rather than isolated positivity to individual molecular allergens, may represent an important feature associated with clinically relevant fish allergy. Given the substantial correlation among several β-parvalbumin components, however, the cumulative burden score should not be interpreted as representing independent sensitization events. Rather, it is likely to reflect the breadth of serologic IgE recognition across closely related β-parvalbumins and therefore may provide an integrated measure of the overall extent of molecular sensitization. This interpretation is consistent with the clustering analyses, which demonstrated closely related co-sensitization patterns among several β-parvalbumins. Consistent with this interpretation, cumulative β-parvalbumin burden showed strong discriminatory performance in our cohort and emerged as one of the most informative markers for distinguishing allergic from tolerant sensitized children.Raj c parvalbumin did not show a significant discriminatory role in our cohort, a finding that is biologically plausible given that Raj c represents an α-parvalbumin, which differs structurally and immunologically from the β-parvalbumins that constitute the major allergens in bony fish (10, 12). Patients allergic to bony fish have been reported to frequently tolerate cartilaginous fish such as ray, likely because α-parvalbumins exhibit substantially lower allergenicity than the β-parvalbumins found in bony fish (23). Moreover, consumption of cartilaginous fish is uncommon in many regions, including Türkiye, which may further limit the clinical relevance of sensitization to Raj c in routine pediatric practice.

Sensitization to Gad m 2_3, comprising β-enolase and aldolase A, represents a distinct molecular sensitization pattern that differs from parvalbumin-driven fish allergy. These proteins are considered minor fish allergens and, unlike parvalbumins, exhibit lower thermal stability and more limited interspecies cross-reactivity (24). In our cohort, sensitization to Gad m 2_3 was observed exclusively among children with clinical fish allergy. Although this finding is clinically intriguing, only six children were sensitized to Gad m 2_3, resulting in considerable statistical uncertainty. Accordingly, Gad m 2_3 should be regarded as an exploratory candidate marker that warrants further investigation rather than as an independently validated predictor of clinical fish allergy. Nevertheless, this observation is consistent with previous component-resolved diagnostic studies suggesting that low-prevalence non-parvalbumin sensitizations may carry clinical relevance when present (14), although their diagnostic value requires further validation.

From a clinical standpoint, an important strength of our study is the well-defined comparison group. All children classified as sensitized but tolerant regularly consumed fish without any history of clinical reactions, supporting the presence of clinical tolerance despite molecular sensitization. In contrast, dietary avoidance was highly restrictive among children with clinical fish allergy. Of the 28 children with a history of fish-related reactions, only 9 (32.1%) reported consuming fish species other than the one that triggered their reaction, whereas 19 children (67.9%) avoided all fish species entirely. This pattern is consistent with previous reports indicating that generalized avoidance of fish is often adopted in clinical practice because of concerns regarding potential cross-reactivity and the limited predictive value of conventional diagnostic tests (7, 8).

In pediatric practice, distinguishing clinically relevant sensitization from asymptomatic sensitization remains challenging. Skin prick testing is limited by variability in commercial fish extracts (25), while OFCs, although considered the diagnostic gold standard, are time-consuming and not always feasible. In this setting, molecular sensitization profiling, particularly when combined with assessment of cumulative β-parvalbumin sensitization burden, may provide additional information for clinical evaluation beyond conventional diagnostic tests.

Recent studies using multiplex component-resolved diagnostics have suggested that molecular sensitization profiles may support more personalized approaches to the evaluation and management of fish allergy (8, 14). Our findings extend this concept by indicating that not only sensitization to individual β-parvalbumins but also the cumulative burden of β-parvalbumin sensitization may be relevant when assessing clinical risk. As the studies cited above were also conducted using the ALEX2 platform, the consistency of these findings should be interpreted within the context of this shared platform. Nevertheless, because multiplex molecular allergy platforms differ in allergen composition, molecular coverage, and analytical characteristics, caution is warranted when extrapolating findings across different platforms. Within this context, molecular allergen profiling, particularly assessment of the cumulative β-parvalbumin sensitization burden, may assist clinicians in identifying selected fish-sensitized children who could benefit from further evaluation, including targeted OFCs, provided that molecular findings are interpreted in conjunction with the clinical history and existing diagnostic work-up. Children with clinical fish allergy were introduced to fish at a slightly later age than sensitized but tolerant children. Although this difference reached statistical significance, the absolute difference was only one month. Therefore, given the observational nature of the study, this finding should be interpreted with caution, and the relationship between age at fish introduction and the development of fish allergy requires further investigation. Prospective studies are needed to clarify whether this association reflects a causal relationship or the influence of other underlying factors.

An additional clinically relevant consideration is the influence of geographic and dietary factors on the interpretation of molecular diagnostic results. Fish consumption patterns vary widely across regions, and the spectrum of clinically relevant fish species differs accordingly. Consequently, the clinical utility of molecular diagnostic panels depends on how well the included allergens reflect locally consumed fish species, highlighting the need for region-specific interpretation of molecular sensitization data. This consideration may be particularly relevant in populations with distinct dietary habits, where the spectrum of fish species associated with allergic reactions may differ from those represented in commercially available molecular diagnostic panels.

This study has several limitations. It was conducted at a single tertiary pediatric allergy referral center and included a relatively small cohort of fish-sensitized children. Consequently, the study population was likely enriched for highly atopic children with multiple sensitizations and may not fully represent community-based fish-sensitized populations, limiting the generalizability of the findings. Although patient classification was partly based on clinical history, this approach reflects routine clinical practice in situations where OFCs are not always feasible. In addition, the proposed models and candidate decision thresholds were developed and internally validated within the same cohort and therefore require external validation in independent populations. Finally, the findings are specific to the ALEX2 platform, and species-specific OFCs or functional studies were not systematically performed. Consequently, the observed co-sensitization patterns should not be interpreted as direct evidence of immunological cross-reactivity.

In conclusion, this study suggests that molecular sensitization profiles provide clinically relevant information beyond the presence of fish-specific IgE alone and may help distinguish clinical fish allergy from asymptomatic sensitization in children. While β-parvalbumins remain the dominant fish allergens, Clu h 1 and Cyp c 1 were the molecular markers most consistently associated with clinical fish allergy. Importantly, allergic children exhibited a substantially greater cumulative β-parvalbumin sensitization burden, supporting the concept that the breadth of molecular sensitization may be associated with clinically relevant fish allergy. Sensitization to Gad m 2_3 was observed only among allergic children in this cohort and should be regarded as a low-prevalence exploratory finding that warrants further investigation rather than an established diagnostic marker, whereas Raj c parvalbumin did not show a significant association with clinical fish allergy. This finding may be related to structural differences between α- and β-parvalbumins as well as the limited consumption of cartilaginous fish in Türkiye. Overall, these findings suggest that molecular allergen profiling, together with assessment of cumulative β-parvalbumin sensitization burden, may contribute to risk stratification among fish-sensitized children. However, the proposed models and candidate decision thresholds require validation in larger independent cohorts before routine clinical implementation.

## Data Availability

The datasets are not publicly available due to ethical and privacy restrictions concerning clinical data from pediatric participants. Any potential data sharing would be subject to applicable data-protection requirements and approval by the relevant institution and ethics committee. Requests to access the datasets should be directed to ST, stutendal@yahoo.com.
