# Supplementary material for: Molecular sensitization profiles and β-parvalbumin sensitization burden associated with clinical fish allergy in children
Source: Front Immunol. 2026 Aug 3;17:1918831. doi: 10.3389/fimmu.2026.1918831 (PMC13477939; doi:10.3389/fimmu.2026.1918831)
Supplement: Supplementary file 1 [file Table1.docx]

**Table S1. Fish species associated with clinical reactions, oral food challenges, and fish consumption patterns in the study cohort**

| Clinical characteristic | Description |
| --- | --- |
| Fish species most commonly implicated in clinical reactions | Sea bass (*Dicentrarchus labrax*), sea bream (*Sparus aurata*), mackerel (*Scomber* spp.), anchovy (*Engraulis encrasicolus*), salmon (*Salmo salar*), and bluefish (*Pomatomus saltatrix*) |
| Fish species most frequently used for oral food challenges (OFCs) | Sea bass (*Dicentrarchus labrax*), sea bream (*Sparus aurata*), salmon (*Salmo salar*), and tuna (*Thunnus* spp.) |
| Fish consumption in sensitized but tolerant children (n = 33) | Regular consumption of multiple fish species without clinical reactions |
| Fish consumption in fish-allergic children (n = 28) | Nineteen children (67.9%) avoided all fish species, whereas nine children (32.1%) continued to consume selected fish species. Among these, tuna was the most frequently tolerated species in three children, while the remaining six tolerated one or two fish species, most commonly anchovy, horse mackerel (*Trachurus* spp.), or mackerel (*Scomber* spp.). |
